# Molecular Proliferation Marker Ki-67 in Breast Cancer: The Role of Vitamin D Status, Hormone Receptor Expression, and Clinicopathological Factors—A Cross-Sectional Study

**DOI:** 10.3390/ijms27198797

**Published:** 2026-10-01

**Authors:** Dorota Weber, Robert Łuczyk, Anna Pacian, Teresa Kulik, Monika Baryła-Matejczuk

**Affiliations:** 1 Faculty of Health Sciences, WSEI University, 20-209 Lublin, Poland; teresa.kulik@wsei.pl; 2 Faculty of Medical and Health Sciences, University of Siedlce, 08-110 Siedlce, Poland; luczykrobert@op.pl; 3 Department of Health Education, Faculty of Nursing Development, Medical University in Lublin, 20-059 Lublin, Poland; anna.pacian@umlub.edu.pl; 4 Institute of Psychology and Pedagogy, WSEI University, 20-209 Lublin, Poland; monika.baryla@wsei.pl

**Keywords:** Ki-67, breast cancer, molecular subtypes, 25-hydroxyvitamin D, estrogen receptor, progesterone receptor, HER2, menopausal status, BMI, immunohistochemistry

## Abstract

Ki-67 is a well-established nuclear biomarker of tumor cell proliferation with key importance for the molecular classification and prognostic stratification of breast cancer. This cross-sectional study included 101 women with histopathologically confirmed invasive breast cancer treated at the St. John of Dukla Oncology Center of the Lublin Region (COZL), Poland (2018–2019). Ki-67 expression was assessed immunohistochemically on formalin-fixed, paraffin-embedded tumor tissue, and independent predictors were identified using multiple linear regression. The median Ki-67 was 20% (IQR: 15–40%). Ki-67 differed significantly between molecular subtypes (Kruskal–Wallis test H = 16.122; *p* = 0.001; η^2^ = 0.138): Luminal A tumors had the lowest Ki-67 (median 18%), whereas the Luminal B, HER2-enriched, and Triple-negative subtypes showed higher values (median 30% each). Ki-67 was significantly lower in tumors expressing the estrogen receptor (*p* = 0.005) and progesterone receptor (*p* = 0.023). Because the Luminal A/B distinction incorporates a Ki-67 threshold by definition, a multivariable model excluding molecular subtype was fitted using ER, PR, HER2, menopausal status, age, BMI category, and 25(OH)D as candidate predictors. This model identified HER2 positivity (b = 11.26; *p* = 0.024) and postmenopausal status (b = 17.93; *p* = 0.017) as independent predictors of higher Ki-67, with a borderline inverse association for age (b = −0.65; *p* = 0.092); the model explained 10.1% of variance (R^2^ = 0.101; F(3,95) = 3.574; *p* = 0.017), and neither ER, PR, BMI, nor 25(OH)D were retained. Serum 25(OH)D concentration showed no meaningful association with Ki-67, either unadjusted (Spearman ρ = 0.035; *p* = 0.730) or when forced into the retained multivariable model regardless of significance (adjusted b = −0.032; 95% CI: −0.337–0.272; *p* = 0.834); no association was observed between circulating 25(OH)D and Ki-67 in this cohort, although indirect biological relationships cannot be excluded.

## 1. Introduction

Breast cancer is the most frequently diagnosed malignancy and the leading cause of cancer death among women worldwide [1]. Precise prognostic stratification is of key importance for optimizing therapeutic decisions and predicting treatment outcomes. Among the molecular determinants of tumor biology, the Ki-67 proliferation index—a non-histone nuclear protein encoded by the *MKI67* gene—has become one of the most clinically informative biomarkers [2]. Ki-67 expression is assessed immunohistochemically and reflects the proportion of tumor cells actively engaged in the cell cycle (G1, S, G2, and M phases), with a complete absence of expression in quiescent cells (G0 phase) [3].

In breast cancer, Ki-67 is a component of the surrogate immunohistochemistry (IHC)-based molecular subtype classification system endorsed by international consensus guidelines. Luminal A tumors, characterized by high hormone receptor expression, the absence of HER2 overexpression, and low Ki-67 (<20%), display a relatively indolent biological course and a favorable prognosis. The Luminal B, HER2-enriched, and Triple-negative (TNBC) subtypes are characterized by higher Ki-67 values, greater genomic instability, and a more aggressive clinical course [4,5]. Beyond its role in classification, Ki-67 provides independent prognostic information and is taken into account when selecting adjuvant chemotherapy [6].

Vitamin D—a lipophilic secosteroid molecule with pleiotropic biological functions—has attracted considerable interest in oncology owing to its antiproliferative, pro-apoptotic, and immunomodulatory properties [7]. Its active metabolite, 1,25-dihydroxyvitamin D [1,25(OH)_2_D], acts via the vitamin D receptor (VDR)—a nuclear transcription factor present in mammary epithelial cells—regulating the expression of genes that control cell cycle progression, differentiation, and apoptosis [8]. Preclinical data indicate that vitamin D signaling suppresses the expression of proliferation-related genes, including *MKI67*, through modulation of the Wnt/β-catenin and EGFR pathways [9]. Epidemiological studies have demonstrated inverse associations between serum 25-hydroxyvitamin D [25(OH)D] concentration—a recognized clinical indicator of vitamin D status—and breast cancer risk, tumor aggressiveness, and unfavorable pathological features [10,11]. Nevertheless, direct clinical evidence linking vitamin D status to Ki-67 expression in a multivariable model accounting for molecular subtype, menopausal status, and metabolic parameters remains limited.

Additional clinicopathological factors—including menopausal status, body mass index (BMI), and age—may independently modulate Ki-67 expression. Breast cancers in postmenopausal women often show higher Ki-67 despite a low-estrogen environment [12]. Obesity, characterized by chronic inflammation and dysregulated adipokine signaling, has been linked to increased tumor proliferative activity [13]. Age at diagnosis may exert a complex, subtype-dependent effect on proliferative activity [14].

The present study had two aims: (1) to characterize the distribution of Ki-67 expression according to molecular subtype, hormone receptor status, and menopausal status in a cohort of Polish women with invasive breast cancer; (2) to identify independent clinicopathological and biochemical predictors of Ki-67 using multiple linear regression, with particular attention to the potential influence of serum 25(OH)D concentration.

Although Ki-67 has been examined in relation to molecular subtype or to vitamin D status in isolation, few studies have jointly modeled hormone receptor status, menopausal status, BMI, and a comprehensive biochemical panel—including 25(OH)D—within a single multivariable framework in a well-characterized Central European cohort, while explicitly avoiding circularity between Ki-67 and Ki-67-derived subtype categories. This integrative, non-circular approach allows the relative contribution of vitamin D status to be evaluated net of clinicopathological confounders and generates a clear, testable finding: that circulating 25(OH)D shows no meaningful association with Ki-67, either unadjusted or after multivariable adjustment, in this cohort.

## 2. Results

### 2.1. Characteristics of the Study Population

The study included 101 women with invasive breast cancer aged 32–71 years (median 51 years; IQR: 46–61). The majority were postmenopausal (59.41%). Normal body weight was recorded in 42.57% of participants, overweight in 39.60%, and obesity in 17.83%. The most common molecular subtypes were Luminal A (40.59%) and Luminal B (31.68%), followed by Triple-negative (16.83%) and HER2-enriched (10.89%). ER expression was found in 78.22% of patients, PR in 69.31%, and HER2 overexpression in 29.70%. The median Ki-67 was 20% (IQR: 15–40%; range 3–80%), and the median 25(OH)D was 26 ng/mL (IQR: 18–36). Low Ki-67 proliferative activity (<15%) was observed in 27.72% of patients, intermediate (15–30%) in 40.59%, and high (>30%) in 29.70%. Participant characteristics are presented in Table 1.

Among the 94 participants with recorded sampling date, blood collection occurred in summer (April–September) for 54 (57.4%) and in winter (October–March) for 40 (42.6%), a reasonably balanced distribution that limits the likelihood of strong seasonal confounding of 25(OH)D measurements, although data on vitamin D supplementation were not systematically available (Section 4.5).

### 2.2. Ki-67 According to Menopausal Status

Ki-67 values were numerically higher in postmenopausal women (median 27.5%; IQR: 15–60%) than in premenopausal women (median 18%; IQR: 12–30%), and a higher proportion of postmenopausal women had Ki-67 > 30% (35.00% vs. 21.95%). However, this difference did not reach statistical significance (Mann–Whitney U test: Z = −1.801; *p* = 0.072; Table 2).

### 2.3. Ki-67 According to Molecular Subtype

**Figure 1:**
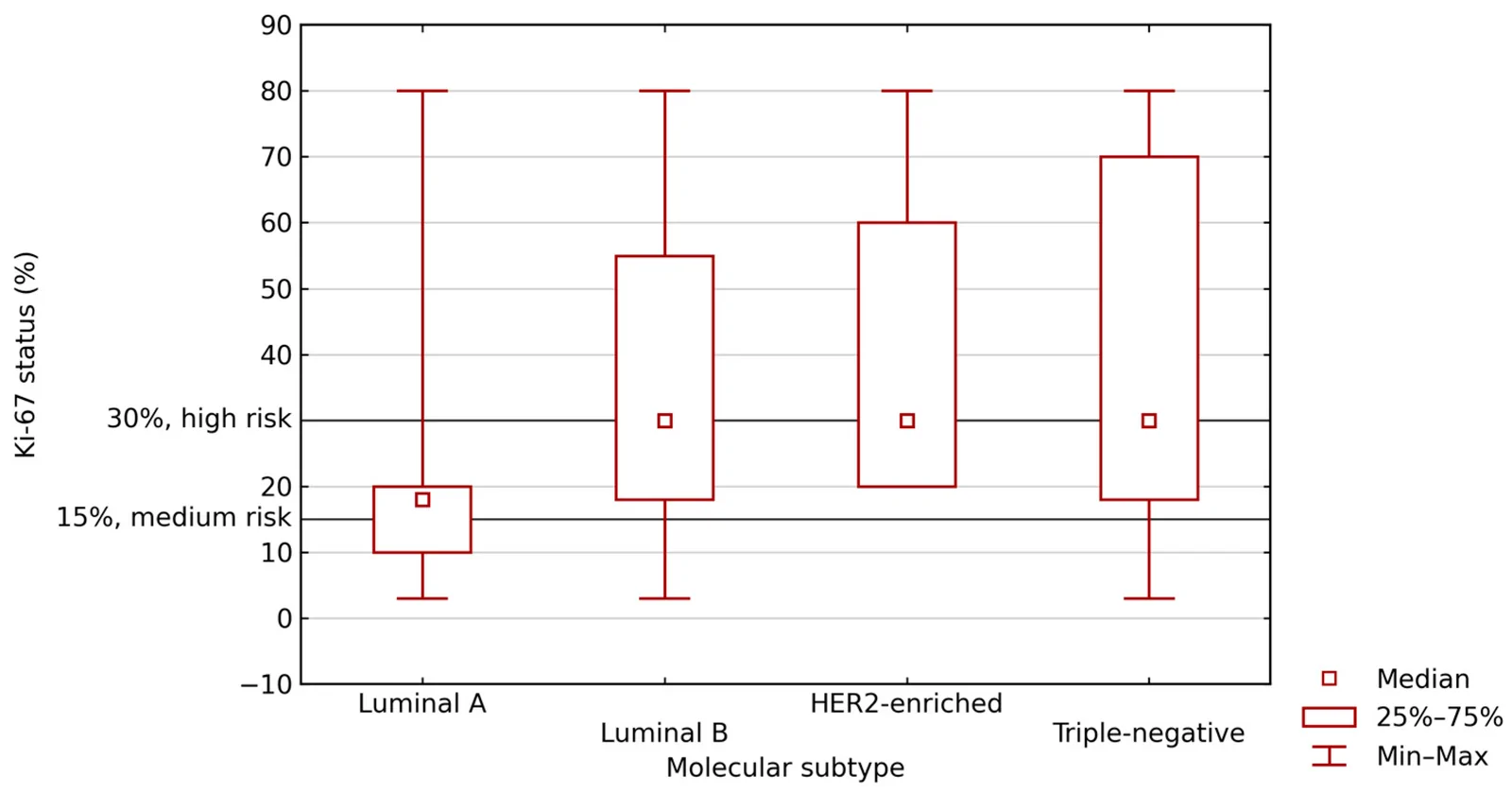
Ki-67 expression (%) according to molecular subtype of breast cancer (Luminal A, Luminal B, HER2-enriched, Triple-negative).

Statistically significant differences in Ki-67 expression were found between molecular subtypes (Kruskal–Wallis test H(3, *n* = 99) = 16.122; *p* = 0.001; η^2^ = 0.138), indicating a large effect size. Pairwise Dunn’s test comparisons showed that Luminal A tumors had significantly lower Ki-67 compared with Luminal B (median 18% vs. 30%; *p* = 0.013), HER2-enriched (30%; *p* = 0.022), and Triple-negative (30%; *p* = 0.035) tumors. No significant differences were found between the remaining subtypes. High Ki-67 (>30%) was most common in the Triple-negative (41.18%) and Luminal B (40.63%) subtypes, and least common in Luminal A (14.63%; Table 3, Figure 1).

### 2.4. Ki-67 According to Hormone Receptor Status

**Figure 2:**
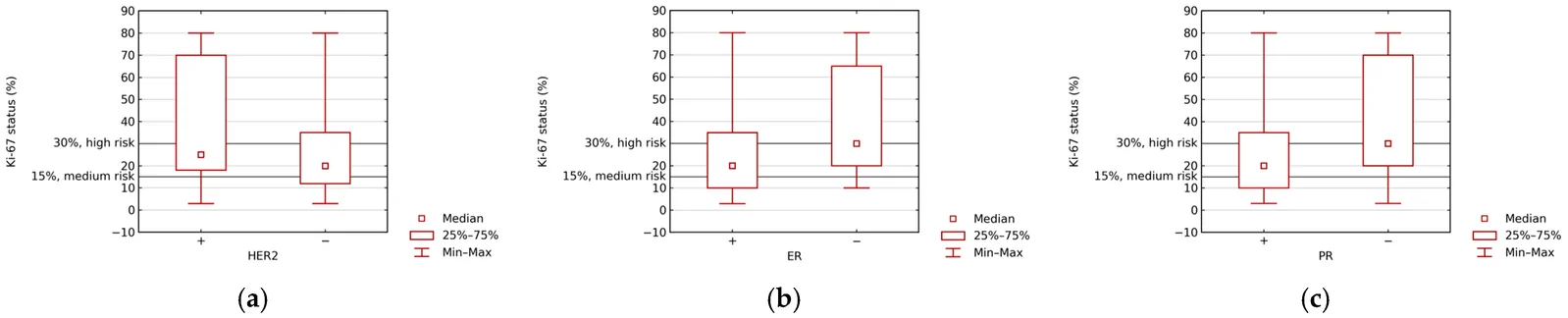
Ki-67 expression (%) according to hormone receptor status: (**a**) HER2; (**b**) estrogen receptor (ER); (**c**) progesterone receptor (PR).

Ki-67 expression was significantly lower in ER-positive tumors (median 20% vs. 30% in ER-negative tumors; Mann–Whitney U test: Z = −2.811; *p* = 0.005) and PR-positive tumors (median 20% vs. 30% in PR-negative tumors; Z = −2.276; *p* = 0.023). HER2 overexpression was associated with numerically higher Ki-67 (median 25% vs. 20%), although this difference did not reach statistical significance (Z = 1.572; *p* = 0.116; Table 4, Figure 2).

### 2.5. Multivariable Predictors of Ki-67

Because the Luminal A/B distinction incorporates a Ki-67 threshold by definition, molecular subtype was excluded from the multivariable model to avoid circularity. Candidate predictors—ER, PR, HER2 status, menopausal status, age, BMI category, and 25(OH)D—were entered into an initial model and reduced via backward stepwise elimination (removal threshold *p* > 0.10). The final model was statistically significant (F(3,95) = 3.574; *p* = 0.017) and explained 10.1% of the variance in Ki-67 (R^2^ = 0.101; adjusted R^2^ = 0.073; standard error of the estimate = 22.461; *n* = 99). Three predictors were retained in the final model (Table 5).

Postmenopausal status was associated with higher Ki-67 (b = 17.929; 95% CI: 3.319–32.539; *p* = 0.017). HER2 positivity was independently associated with higher Ki-67 (b = 11.258; 95% CI: 1.491–21.024; *p* = 0.024), consistent with the known biology of HER2-driven tumors. Older age showed a borderline inverse association with Ki-67 (b = −0.646; 95% CI: −1.399–0.107; *p* = 0.092). ER, PR, BMI category, and 25(OH)D were not retained in the final model (all *p* > 0.10); in particular, neither overweight nor obesity remained associated with Ki-67.

Serum 25(OH)D concentration was not retained as an independent predictor of Ki-67 in the model, consistent with the unadjusted analysis (Spearman ρ = 0.035; 95% CI: −0.163–0.231; *p* = 0.730), which showed no meaningful correlation between 25(OH)D and Ki-67 across the full range of vitamin D status. Ki-67 was also examined across clinically relevant 25(OH)D categories (deficient, <20 ng/mL; insufficient, 20–29 ng/mL; sufficient, ≥30 ng/mL); median Ki-67 was identical (20%) across all three categories (deficient: *n* = 27; insufficient: *n* = 29; sufficient: *n* = 43; Kruskal–Wallis H = 0.805; *p* = 0.669), providing no evidence of a threshold effect. Because 25(OH)D is the primary variable of interest, its adjusted association with Ki-67 is also reported directly, independent of statistical significance, by forcing 25(OH)D into the retained model (menopausal status, HER2, age) rather than inferring its effect from its removal during stepwise selection: b = −0.032 (95% CI: −0.337–0.272; standardized β = −0.021; *p* = 0.834; four-predictor model R^2^ = 0.102, adjusted R^2^ = 0.064, F(4,94) = 2.665, *p* = 0.037, standard error of the estimate = 22.575, *n* = 99), confirming a negligible, non-significant adjusted effect consistent with the unadjusted correlation above. The Durbin–Watson statistic (d = 1.55) indicated a slight positive autocorrelation of residuals, without a substantial violation of the independence-of-errors assumption. Variance inflation factors were below 3 for all retained predictors (menopausal status and age: VIF = 2.58; HER2: VIF = 1.00), indicating no problematic multicollinearity, including between age and menopausal status.

Because Ki-67 was not normally distributed, the adequacy of the linear model was formally evaluated using residual diagnostics rather than assumed. The Shapiro–Wilk test applied to model residuals indicated departure from normality (W = 0.921; *p* < 0.001; residual skewness = 0.74), and the Breusch–Pagan test indicated mild heteroscedasticity (LM = 8.35; *p* = 0.039). Cook’s distance identified six observations exceeding the conventional 4/*n* threshold (maximum Cook’s D = 0.062), but no observations exceeded twice the mean leverage, indicating no single point exerted undue influence. Given these assumption violations, the three retained predictors were re-estimated using heteroscedasticity-robust (HC3) standard errors and confirmed using case-resampling bootstrap (5000 replicates, percentile 95% CI): HER2 (HC3 *p* = 0.044; bootstrap 95% CI: 1.20–22.52) and postmenopausal status (HC3 *p* = 0.015; bootstrap 95% CI: 3.63–32.17) remained significant, while age remained borderline/non-significant (HC3 *p* = 0.107; bootstrap 95% CI: −1.39–0.13, including zero), confirming that the principal findings are robust to the observed departures from normality and homoscedasticity.

To assess the influence of the two participants with missing Ki-67 (Section 4.2), a sensitivity analysis was performed in which Ki-67 was imputed for these participants using the median value for their assigned molecular subtype (Triple-negative; both participants had ER−/PR−/HER2− status, so subtype assignment did not depend on Ki-67). This single, exploratory imputation is presented only as a robustness check and not as the primary analysis. In this sensitivity dataset (*n* = 101), the unadjusted correlation between 25(OH)D and Ki-67 remained essentially null (Spearman ρ = 0.030; 95% CI: −0.167–0.224; *p* = 0.768), and the multivariable model retained the same three predictors with materially unchanged estimates (HER2: b = 11.38, *p* = 0.021; menopausal status: b = 17.43, *p* = 0.016; age: b = −0.62, *p* = 0.095; R^2^ = 0.100), indicating that the primary complete-case results (*n* = 99) are robust to this pattern of missing data.

## 3. Discussion

This study characterized the distribution of Ki-67 and its clinical, molecular, and biochemical determinants in a cohort of Polish women with invasive breast cancer. The principal observations are as follows: (1) Ki-67 differed markedly across molecular subtypes, with Luminal A showing the lowest proliferative activity—an association that is expected given that Ki-67 status contributes to the definition of the Luminal A/B distinction, and that is therefore reported here as a descriptive comparison rather than as evidence of an independent predictive relationship (see below); (2) Ki-67 was significantly lower in ER-positive and PR-positive tumors on unadjusted comparison, although ER and PR were not retained in a multivariable model constructed without the circular subtype term and cannot be described as independently associated with Ki-67; (3) in a corrected multivariable model excluding molecular subtype, postmenopausal status and HER2 positivity were independent predictors of higher Ki-67, with a borderline inverse association for age, whereas BMI category was not retained, indicating that the originally reported obesity association was not robust to this correction; (4) serum 25(OH)D concentration showed no meaningful unadjusted correlation with Ki-67 (Spearman ρ = 0.035; *p* = 0.730) and was not retained as an independent predictor in the corrected multivariable model.

The association between molecular subtype and Ki-67 observed in our cohort (η^2^ = 0.138) is consistent with extensively published data and reflects the biological basis of the IHC classification system [4,5]. This large univariate effect size is, however, expected rather than fully independent evidence, because Ki-67 status (<20% vs. ≥20%) contributes directly to the definition of the Luminal A/B distinction; the comparison across subtypes is therefore partly circular and is reported here as a descriptive characterization of the cohort rather than as proof that molecular subtype independently or dominantly determines Ki-67. The HER2-enriched and Triple-negative subtypes, whose definitions do not depend on the Ki-67 threshold, showed Ki-67 values numerically similar to Luminal B, which lends some independent support to a genuine association between aggressive, non-luminal phenotypes and higher proliferative activity; this pattern is consistent with reports that immunohistochemical surrogates relying on Ki-67 poorly distinguish Luminal A from Luminal B tumors when benchmarked against gene expression-based subtyping [15,16]. Nonetheless, definitive separation of this effect from the circularity inherent in the Luminal A/B classification would require a reanalysis using tumor groupings that do not incorporate Ki-67 (e.g., ER/PR/HER2-based categories alone), which we identify as a priority for future work. Clinically, elevated Ki-67 remains a factor considered when planning neoadjuvant chemotherapy [6], independent of how it relates to subtype classification.

The significant inverse associations between Ki-67 and ER/PR expression reflect the well-established biology of hormone receptor signaling in the regulation of the breast tumor cell cycle. Estrogen receptor activation promotes cyclin D1 expression, yet ER-positive tumors paradoxically show lower Ki-67, because the ER-positive phenotype is inherently linked to a less aggressive biology [17]. This complexity underscores the importance of multivariable modeling.

The independent association between postmenopausal status and higher Ki-67, observed after adjustment for subtype, is a clinically relevant finding. Contrary to the assumption that a high-estrogen premenopausal environment drives proliferation, breast cancers in postmenopausal women in our cohort showed higher Ki-67. This may reflect dysregulated signaling through increased aromatase activity in adipose tissue, altered IGF-1 signaling, and higher rates of de novo lipogenesis in postmenopausal women [12,13].

The univariate association between obesity and higher Ki-67 (Table 1) was not confirmed as an independent predictor once molecular subtype was excluded from the multivariable model (Section 2.5), suggesting that this association may have been confounded by the distribution of molecular subtypes across BMI categories rather than reflecting a robust, subtype-independent biological relationship. A direct effect of adiposity on proliferation—for example via adipose tissue-derived leptin, insulin, IGF-1, and pro-inflammatory cytokines acting on the PI3K/Akt/mTOR and JAK-STAT3 pathways—remains biologically plausible [13] and could regulate transcription of *MKI67* [18], but this mechanism was not supported by the corrected multivariable analysis and should be regarded as hypothesis-generating rather than confirmed. HER2 positivity, in contrast, was independently associated with higher Ki-67 in the corrected model, consistent with the established role of HER2-driven signaling in promoting cell cycle progression, and represents a more robust finding from this dataset.

The absence of an independent association between serum 25(OH)D concentration and Ki-67 in the multivariable model is an important negative finding. Although vitamin D signaling exhibits antiproliferative properties in preclinical breast cancer models—including suppression of *MKI67* expression via VDR-mediated mechanisms [8,9]—the translation of these effects into clinical settings is complex, in part because circulating 25(OH)D reflects systemic vitamin D supply rather than intratumoral VDR expression or local vitamin D metabolism, which can diverge from serum levels [19,20]. In the present cohort, the unadjusted correlation between 25(OH)D and Ki-67 was essentially null (Spearman ρ = 0.035; 95% CI: −0.163–0.231; *p* = 0.730), and no association emerged in the corrected multivariable model excluding molecular subtype either. This differs somewhat from reports by Jacobs et al. [21] and Hatse et al. [22], who described an attenuation of the vitamin D–tumor aggressiveness relationship after adjustment for molecular subtype, implying a detectable unadjusted association; here, no unadjusted association was observed at all, suggesting that any relationship between circulating 25(OH)D and proliferative activity, if present, is weaker than previously reported and may not be reliably detectable in a single-center cohort of this size. These findings should be considered alongside other studies reported in the literature [23,24,25,26,27]. Given the cross-sectional design of the present study, this association should not be interpreted as evidence of a causal effect of vitamin D status on tumor aggressiveness.

Several limitations should be noted. The cross-sectional design precludes causal inference. The modest explained variance of the corrected multivariable model (R^2^ = 0.101) indicates a substantial contribution of unmeasured biological factors. The single-center design and Polish population may limit generalizability. IHC assessment of Ki-67 is subject to inter-laboratory variability, although procedures consistent with ASCO/CAP guidelines were used. The value of d = 1.55 indicates a slight autocorrelation of residuals. A further important limitation is that the Luminal A/B classification incorporates a Ki-67 threshold by definition, so comparisons of Ki-67 across these two subtypes, and their inclusion as predictors in the multivariable model, are partly circular and should be interpreted descriptively rather than as independent predictive evidence; a reanalysis using tumor groupings that do not incorporate Ki-67 is planned. Additional limitations include potential Ki-67 scoring variability related to hotspot selection, tumor heterogeneity, and inter-observer reproducibility, which were not formally quantified in this study but are increasingly well documented in recent standardization efforts [28,29]; the 2–8 week postoperative timing of 25(OH)D sampling, which may not reflect preoperative or long-term vitamin D status and for which data on supplementation and treatment before sampling were not systematically available (although season of blood collection was recorded and was reasonably balanced; Section 2.1); the absence of tumor-level measures of VDR expression or local vitamin D metabolism, precluding any inference about intratumoral vitamin D signaling; the modest sample size, particularly for subgroup comparisons; and residual confounding from unmeasured variables. An initially recorded outlier value for vitamin B12 (14,054 pg/mL) was identified during data verification as a database entry error and has been corrected (Table 1) following review of the primary laboratory records; the corrected cohort maximum is 4123 pg/mL. The cross-sectional design cannot establish temporality and should not be interpreted as demonstrating causality for any of the reported associations. Nonetheless, by simultaneously accounting for hormonal and clinicopathological covariates within a single, non-circular model, this study provides a more granular and methodologically sound correlational framework than previously available for this population, clarifying that circulating 25(OH)D shows no meaningful association with Ki-67 in this cohort and identifying HER2 status and menopausal status as the more robust correlates warranting confirmation in future prospective, mechanistic studies.

## 4. Materials and Methods

### 4.1. Study Design, Setting, and Ethical Approval

This cross-sectional observational study was conducted at the St. John of Dukla Oncology Center of the Lublin Region (COZL) in Lublin, Poland, between 2018 and 2019. The study protocol was approved by the Bioethics Committee (Resolution No. KE-0254/78/2019) and conducted in accordance with the Declaration of Helsinki. Written informed consent was obtained from all participants prior to enrollment.

### 4.2. Participants

Inclusion criteria were: (1) histopathologically confirmed invasive breast cancer; (2) completed surgical treatment; (3) availability of a complete IHC panel (ER, PR, HER2, Ki-67); (4) age ≥ 18 years. A total of 101 patients were enrolled. Sample sizes therefore varied slightly across analyses: *n* = 101 for the descriptive characteristics reported in Table 1, and *n* = 99 for all comparative and multivariable analyses (Table 2, Table 3, Table 4 and Table 5), reflecting the exclusion of two patients with missing Ki-67 assessment; both had valid, recorded ER-negative/PR-negative/HER2-negative (Triple-negative) status, so their exclusion affected only Ki-67-dependent analyses and did not affect subtype assignment. The applicable sample size is stated in the heading of each table.

### 4.3. Immunohistochemical Assessment of Ki-67

Ki-67 expression was assessed immunohistochemically on FFPE tissue using the MIB-1 clone antibody (Dako/Agilent, Santa Clara, CA, USA), in accordance with the guidelines of the International Ki67 in Breast Cancer Working Group [30]. Ki-67 was expressed as the percentage of positively stained tumor nuclei among at least 500 invasive tumor cells counted in representative fields at ×400 magnification. Scoring used the global assessment method recommended by the Working Group and endorsed in subsequent standardization efforts, which has shown improved inter-laboratory concordance over hotspot-based scoring [28,29]. For descriptive purposes, Ki-67 values were additionally stratified into three proliferative activity levels: low (<15%, with <10% denoting minimal activity), intermediate (15–30%), and high (>30%). These descriptive categories are distinct from, and were not used to derive, the 20% Ki-67 threshold applied to distinguish Luminal A from Luminal B tumors (Section 4.4).

### 4.4. Classification of Molecular Subtypes and Hormone Receptors

ER, PR, and HER2 status were determined by IHC. Hormone receptor positivity was defined as nuclear staining in ≥1% of invasive tumor cells (ASCO/CAP 2020) [31]. HER2 IHC 2+ results were verified by FISH. Subtypes were classified as: Luminal A (ER+ and/or PR+, HER2−, Ki-67 < 20%), Luminal B (ER+ and/or PR+, HER2+ or HER2− with Ki-67 ≥20%), HER2-enriched (ER−, PR−, HER2+), and Triple-negative (ER−, PR−, HER2−).

We note that, because the Luminal A/B distinction incorporates a Ki-67 threshold (<20% vs. ≥20%) by definition, comparisons of Ki-67 across these two subtypes are not statistically independent of subtype assignment. Results involving the Luminal A/B distinction (Section 2.3 and Section 2.5) are therefore reported and interpreted as descriptive characterizations of the cohort rather than as evidence that molecular subtype independently predicts Ki-67; this circularity does not apply to the HER2-enriched and Triple-negative categories, whose definitions do not incorporate Ki-67. This concern is consistent with broader evidence that Ki-67-based immunohistochemical surrogates show only poor-to-moderate concordance with gene expression-based (PAM50) intrinsic subtyping, particularly for distinguishing Luminal A from Luminal B tumors [15,16].

### 4.5. Biochemical and Clinical Data

Blood samples were collected in the morning (08:00–11:00), after an overnight fast, within 2–8 weeks of surgical treatment. Serum 25(OH)D concentration was determined by ECLIA (Vitamin D Total Test, Roche Diagnostics, Mannheim, Germany; Cobas e410 analyzer); intra-assay CV < 6.5%, inter-assay CV < 10%. Fasting glucose, albumin, potassium, calcium, magnesium, total cholesterol, HDL, LDL, and vitamin B12 were also measured. BMI was categorized according to WHO criteria. Menopausal status was defined as the absence of menstruation for ≥12 months or a history of bilateral oophorectomy. The season of blood collection (summer, April–September, vs. winter, October–March) was recorded for a subset of participants (*n* = 94; 54 summer, 40 winter) and is reported descriptively in Section 2.1; data on vitamin D supplementation, pre-sampling treatment, and precise month of collection were not systematically available for the full cohort and are noted as a limitation.

### 4.6. Statistical Analysis

Analyses were performed using Statistica 14 (Cloud Software Group, Inc., Tulsa, OK, USA, 2023). The Shapiro–Wilk test confirmed a non-normal distribution for Ki-67 (*p* < 0.05). Differences between subtypes were assessed using the Kruskal–Wallis test with pairwise Dunn’s test comparisons (Bonferroni correction); effect size η^2^. Dichotomous comparisons were performed using the Mann–Whitney U test. Predictors of Ki-67 were identified using multiple linear regression with backward stepwise elimination (removal threshold *p* > 0.10, matching the criterion applied throughout Section 2.5). Although Ki-67 was not normally distributed, multiple linear regression was used rather than a nonparametric alternative because it allowed simultaneous adjustment for several categorical and continuous covariates and yielded readily interpretable, clinically meaningful effect estimates (unstandardized b coefficients); the adequacy of this approach was subsequently verified through residual and multicollinearity diagnostics (Section 2.5). Descriptive statistics, group comparisons, and the multivariable regression models reported in Table 5 and Section 2.5 (including the model in which 25(OH)D was forced in regardless of significance) were computed in Statistica 14. Supplementary residual normality (Shapiro–Wilk), heteroscedasticity (Breusch–Pagan), influence (Cook’s distance), heteroscedasticity-robust (HC3), and bootstrap diagnostics (Section 2.5) were additionally computed in Python (SciPy 1.11, statsmodels 0.14) applied to the same model residuals, to supplement assumption-checking beyond Statistica’s standard output; because these are closed-form or resampling-based statistical procedures applied to identical data and model specifications, results are software-independent. The significance level was set at α = 0.05. Because the Luminal A/B molecular subtype category is partly defined by a Ki-67 threshold, molecular subtype was excluded a priori from the multivariable regression model to avoid circularity; instead, a clinically prespecified candidate set—ER, PR, HER2 status, menopausal status, age, BMI category, and 25(OH)D—was used, consistent with recommendations to prioritize non-circular, clinically motivated predictors over exhaustive variable selection in modest-sized samples. The direct, unadjusted association between 25(OH)D and Ki-67 was additionally assessed using Spearman’s rank correlation. All primary analyses used complete-case data; a sensitivity analysis with single median-subtype imputation for participants missing Ki-67 was performed to assess potential bias from missingness (Section 2.5).

## 5. Conclusions

This cross-sectional study demonstrates that molecular subtype—particularly Luminal B and Triple-negative—is associated with Ki-67 expression in invasive breast cancer on descriptive comparison (η^2^ = 0.138); because Ki-67 status contributes to the definition of the Luminal A/B distinction, this association is partly circular and should not be interpreted as evidence that subtype independently or dominantly determines Ki-67. In a corrected multivariable model excluding the circular Luminal A/B subtype term, postmenopausal status and HER2 positivity independently contributed to higher Ki-67, with a borderline inverse association for age; obesity was not confirmed as an independent predictor once this correction was applied. On unadjusted comparison, ER and PR positivity were associated with lower proliferative activity, consistent with well-established tumor biology, although neither was retained in the final multivariable model. Serum 25(OH)D concentration showed no association with Ki-67 in this cohort, whether assessed directly (adjusted b = −0.032; 95% CI: −0.337–0.272; *p* = 0.834) or via unadjusted correlation (Spearman ρ = 0.035; *p* = 0.730); this finding does not support a role for circulating vitamin D status as a determinant of proliferative activity in this population. Whether alternative pathways—for example, tumor-level VDR expression or local vitamin D metabolism, which were not measured here—might relate to tumor biology remains an open question for future, hypothesis-driven prospective research rather than a conclusion supported by the present data. These findings underscore the need for a multifactorial approach—encompassing obesity control, menopausal health, and vitamin D status—in the oncological care of breast cancer patients. By integrating clinicopathological, hormonal, and biochemical determinants of Ki-67 within a single multivariable model, this study extends beyond previous single-domain analyses and provides a concrete basis for hypothesis-driven, prospective research into the mechanisms linking vitamin D status, tumor phenotype, and proliferative activity.

## Figures and Tables

**Table 1:** Demographic, clinical, and biochemical characteristics of study participants (*n* = 101).

Variable	Me	Q1	Q3	Min–Max
Age (years)	51	46	61	32–71
Ki-67 (%)	20	15	40	3–80
25(OH)D (ng/mL)	26	18	36	8–118
Glucose (mg/dL)	96	89	102	57–147
Albumin (g/dL)	4.28	4.07	4.46	3.66–4.80
Potassium (mEq/L)	4.25	3.97	4.63	2.42–5.68
Calcium (mmol/L)	2.38	2.30	2.45	2.08–4.40
Magnesium (mmol/L)	2.01	1.91	2.13	1.04–2.41
Total cholesterol (mg/dL)	214	181	242	114–364
HDL cholesterol (mg/dL)	58	52	68	25–93
LDL cholesterol (mg/dL)	126.5	102	154.5	54–254
Vitamin B12 (pg/mL)	341	261	460	79–4123
		***n***	**%**	
BMI	Normal weight (18.5–24.9)	43	42.57	
	Overweight (25.0–29.9)	40	39.60	
	Obesity (≥30.0)	18	17.83	
Menopausal status	Premenopausal	41	40.59	
	Postmenopausal	60	59.41	
Molecular subtype	Luminal A	41	40.59	
	Luminal B	32	31.68	
	HER2-enriched	11	10.89	
	Triple-negative	17	16.83	
HER2	+	30	29.70	
	−	71	70.30	
ER	+	79	78.22	
	−	22	21.78	
PR	+	70	69.31	
	−	31	30.69	
Ki-67 category	<15%	28	27.72	
	15–30%	41	40.59	
	>30%	30	29.70	

Me—median; IQR—interquartile range (Q1–Q3); BMI—body mass index; ER—estrogen receptor; PR—progesterone receptor; HER2—human epidermal growth factor receptor 2; 25(OH)D—25-hydroxyvitamin D.

**Table 2:** Ki-67 expression according to menopausal status (*n* = 99).

Menopausal Status	*n*	Me	Q1–Q3	Ki-67 > 30%, *n* (%)	*p*
Premenopausal	41	18	12–30	9 (21.95%)	
Postmenopausal	58	27.5	15–60	21 (35.00%)	
Overall	99	20	15–40	30 (29.70%)	0.072

Mann–Whitney U test: Z = −1.801; *p* = 0.072.

**Table 3:** Ki-67 expression according to molecular subtype of breast cancer (*n* = 99).

Molecular Subtype	*n*	Me	Q1–Q3	Ki-67 > 30%, *n* (%)	Post-Hoc
Luminal A	41	18	10–20	6 (14.63%)	a
Luminal B	32	30	18–55	13 (40.63%)	b
HER2-enriched	11	30	20–60	4 (36.36%)	b
Triple-negative	15	30	18–70	7 (41.18%)	b
Overall	99	20	15–40	30 (29.70%)	

Kruskal–Wallis test: H(3, *n* = 99) = 16.122; *p* = 0.001; η^2^ = 0.138. Different superscript letters (a, b) indicate significant pairwise differences (Dunn’s test): Luminal A vs. Luminal B *p* = 0.013; vs. HER2-enriched *p* = 0.022; vs. Triple-negative *p* = 0.035.

**Table 4:** Ki-67 expression according to hormone receptor status (*n* = 99).

Receptor	Status	*n*	Me	Q1–Q3	Ki-67 > 30%, *n* (%)	*p*
HER2	+	30	25	18–70	12 (40.00%)	0.116
	−	69	20	12–35	18 (25.35%)	
ER	+	79	20	10–35	21 (26.58%)	0.005
	−	20	30	20–65	9 (40.91%)	
PR	+	70	20	10–35	18 (25.71%)	0.023
	−	29	30	20–70	12 (38.71%)	

Mann–Whitney U test. ER+/−: Z = −2.811; *p* = 0.005. PR+/−: Z = −2.276; *p* = 0.023. HER2+/−: Z = 1.572; *p* = 0.116.

**Table 5:** Multiple linear regression model of Ki-67 predictors, excluding molecular subtype to avoid circularity with the Ki-67-based Luminal A/B definition (*n* = 99).

Predictor	β	SE β	b	SE b	t(95)	*p*-Value
Intercept			50.349	16.951	2.970	0.004
Menopausal status (post vs. pre)	0.380	0.156	17.929	7.359	2.436	0.017
HER2 status (+ vs. −)	0.223	0.097	11.258	4.920	2.288	0.024
Age (years)	−0.266	0.156	−0.646	0.379	−1.702	0.092
25(OH)D (forced into retained model) ᵃ	−0.021	0.099	−0.032	0.153	−0.210	0.834

Three-predictor model: R = 0.318; R^2^ = 0.101; adjusted R^2^ = 0.073; F(3,95) = 3.574; *p* = 0.017; standard error of the estimate = 22.461; *n* = 99. β—standardized regression coefficient; SE—standard error; b—unstandardized coefficient. Reference categories: premenopausal; HER2-negative. Candidate predictors entered into the initial model were ER, PR, HER2, menopausal status, age, BMI category (overweight/obesity vs. normal weight), and 25(OH)D; ER, PR, BMI, and 25(OH)D were not retained after backward elimination (*p* > 0.10). ᵃ Not part of the backward-elimination model above; reported separately by forcing 25(OH)D into the three retained predictors (menopausal status, HER2, age) to provide a direct adjusted estimate independent of stepwise selection, per reviewer request (four-predictor model: R^2^ = 0.102, adjusted R^2^ = 0.064, F(4,94) = 2.665, *p* = 0.037, standard error of the estimate = 22.575).

## Data Availability

The data presented in this study are available on request from the corresponding author. The data are not publicly available due to privacy protection of the participants.
